# United, can we be stronger? Did French general practitioners in multi-professional groups provide more chronic care follow-up during lockdown?

**DOI:** 10.1186/s12913-022-07937-z

**Published:** 2022-04-19

**Authors:** Anna Zaytseva, Pierre Verger, Bruno Ventelou

**Affiliations:** 1 Aix-Marseille University, CNRS, EHESS, Centrale Marseille, AMSE, 5-9 Boulevard Maurice Bourdet, CS 50498, 13205, Marseille Cedex 1, France; 2 Southeastern Regional Health Observatory, ORS Paca, Provence-Alpes-Côte d’Azur, Marseille, France, 27 Boulevard Jean Moulin, 13385 Marseille Cedex 5, France; 3 Aix-Marseille University, IRD, AP-HM, SSA, VITROME, IHU-Méditerranée Infection, 19-21 Boulevard Jean Moulin, 13005 Marseille, France

**Keywords:** COVID-19, General practitioners, France, Provider-sponsored organizations, Long-term care

## Abstract

**Background:**

Given the importance of the continuous follow-up of chronic patients, we evaluated the performance of French private practice general practitioners (GPs) practicing in multi-professional group practices (MGP) regarding chronic care management during the first Covid-19 lockdown in Spring 2020 compared to GPs not in MGP. We consider two outcomes: continuity of care provision for chronic patients and proactivity in contacting these patients.

**Methods:**

The cross-sectional web questionnaire of 1191 GPs took place in April 2020. We exploit self-reported data on: 1) the frequency of consultations for chronic patients during lockdown compared to their “typical” week before the pandemic, along with 2) GPs’ proactive behaviour when contacting their chronic patients. We use probit and bivariate probit models (adjusted for endogeneity of choice of engagement in MGP) to test whether GPs in MGP had significantly different responses to the Covid-19 crisis compared to those practicing outside MGP.

**Results:**

Out of 1191 participants (response rate: 43.1%), around 40% of GPs were female and 34% were younger than 50 years old. Regression results indicate that GPs in MGP were less likely to experience a drop in consultations related to complications of chronic diseases (− 45.3%). They were also more proactive (+ 13.4%) in contacting their chronic patients compared to their peers practicing outside MGP.

**Conclusion:**

We demonstrate that the MGP organisational formula was beneficial to the follow-up of patients with chronic conditions during the lockdown; therefore, it appears beneficial to expand integrated practices, since they perform better when facing a major shock. Further research is needed to confirm the efficiency of these integrated practices outside the particular pandemic setup.

**Supplementary Information:**

The online version contains supplementary material available at 10.1186/s12913-022-07937-z.

## Introduction

The Covid-19 pandemic and its subsequent health system responses has already had considerable consequences on the most vulnerable populations. Chronic patients in particular have been severely affected by the pandemic [1–3]. Besides, for the past 20 years, it has been shown that the evolution of primary care is one of the key components of health systems in better preventing and managing chronic patients [4]. The integration of primary care practices, in particular, has been identified as a plausible determinant of a “good” chronic care model, as it allows a more efficient combination of healthcare resources [5, 6]. This paper investigates how general practitioners’ GP participation in multi-disciplinary practices affects the follow-up of their chronic care patients in France.

In France, self-employed general practitioners (GP) ensure more than 90% of primary care. As in other developed countries, a clear trend towards the integration of primary care providers is observed: in a 2010 survey, 54% of GPs indicated practicing in a group; a figure which rose to 61% in 2019 [7]. However, most of these group practices only share premises, and/or non-clinical and back-office functions. The latest, most advanced form of integrated healthcare teams in France is the so-called multi-professional primary care group practice (MGP) [in French: *“maison de santé pluridisciplinaire”*]. MGP are created on a voluntary basis. They provide primary care for the local population and may take part in public health, prevention, health education and the social activities of their choice. All these activities form a “health project” that is signed by all the MGP team members and reported to regional health authorities as proof of coordinated care. This health project usually grants access to some funding in order to facilitate the foundation of the MGP; the conditions of access and the amount varying according to the region. The MGP is required to have at least two full-time equivalent GPs and one paramedic. This institutional cooperation with paramedics is the main novelty for the French ambulatory care system, since this type of cooperation is not mandatory in the primary care system (projects that include cooperation between GPs and hospitals are not eligible). While the minimal requirement on the number of MGP members is two full-time equivalent GPs and one paramedic, on average there were 3.4 different paramedics and 0.5 specialists as well as 3.8 full-time equivalent GPs (data in our sample). The policy was launched in 2007 and has proven to be extremely popular among physicians: in 2020, more than 1300 MGP were actively operating compared to less than 20 in 2008 [8]. However, few French studies really investigate the gains related to integrated practices in terms of the quality of care [9, 10], since the impact of MGP cannot be studied in the framework of a randomized study. They have demonstrated some efficiency gains in terms of quantity of care delivered, e.g. longer patient lists and more home and office visits [9, 10]. The same lack of evidence is observed in the international literature [11, 12]. In the rest of this paper, we will consider GPs’ participation in MGP as our variable of interest indicating an (institutional) marker of integrated practices.

This article aims at evaluating how GPs in MGP perform regarding chronic care management in response to a major shock. Continuous follow-up of chronic patients being essential for better quality of care, the current pandemic, beyond the calamity it represents, provides an opportunity to examine how GPs in MGP can adapt their practices under these unusual conditions. Using an appropriate statistical model, we compare GPs’ performance in MGP, with their counterparts practicing outside MGP, regarding the frequency of consultations for chronic patients as well as their proactivity in contacting these patients during the first lockdown.

## Materials and methods

### Study population

We used data from the national panel of French self-employed GPs, set up in 2018 [13].

GPs were randomly selected from a French exhaustive database of health professionals as of January 1, 2018 and who have signed an informed consent at inclusion (in 2019) to answer six future cross-sectional surveys (one every 9 months). Sampling was stratified for gender, age, workload (annual number of office and home visits; in terciles) and practice location in low GP density municipalities. The panel is representative of GPs practicing in France (excluding Mayotte). GPs planning to retire or to move before the end of data collection, those exclusively practicing alternative medicine as well as those with few gatekeeping duties (fewer than 200 patients) were excluded. The sample benefits from the French “public statistics” label of the National Authority for Statistical Information (*Conseil National de l’Information Statistique*).

### Procedure and questionnaire

At inclusion in 2019, professional investigators contacted GPs to ask them to participate, obtain their consent, and verify inclusion criteria; they then conducted the inclusion interview, collecting information about GPs’ professional characteristics, using computer-assisted telephone interview (CATI) software.

A special Covid-19 cross-sectional survey was decided in March 2020 in order to study GP practices in the face of the pandemic. In line with the prior systematic collection of longitudinal data planned in 2019, an ad-hoc committee was formed at the beginning of the first lockdown in France in order to elaborate short-form surveys focusing on the GPs’ response to the COVID-19 crisis. The committee was comprised of various public health scientists (epidemiologists, sociologists, and health economists), already trained in surveys among GPs. Since there was no past experience of lockdowns, the questionnaire was not based on standardised questions, although it was inspired by literature dealing with the H1N1 crisis [14]. We pilot-tested the questionnaire for clarity, length, and face validity among 6 GPs and modified the wording of several questions found to be unclear.

The web-survey took place between April 9 and April 21 2020; 1191 GPs out of 2761 contacted (43.1%) responded. Since the eligibility criteria were verified at inclusion (in 2019), all the remaining GPs were eligible to participate in the survey in 2020.

We exploit the part of the questionnaire focused on the impact of the lockdown on GPs’ activity (the week before the survey compared to a “typical” week before the pandemic). A “don’t know” answer was also included in each item of the questionnaire.

We used an indicator variable of the intensity of the Covid-19 pandemic at the *département* level (France is divided into 18 regions that are further subdivided into 101 *départements*; each *département* belongs to only one region). This indicator was constructed by the Directorate for Research, Studies, Assessment and Statistics (DREES, French Ministry of Health) from National Institute of Statistics and Economic Studies (Insee) Covid-19 mortality data collected between March 1 and April 20 2020 [15]. We used a dummy variable to isolate the most affected *départements*, where an average change in excess mortality rate was 110.5%.

In addition, we used a set of five dummy variables regarding motivations for the choice of the current practice location from Round 1 of the survey that took place from October 2018 to April 2019. The GPs had to choose between the items related to 1) healthcare services available in the area, 2) the possibility of creating or joining a group practice, 3) the search for an area with low GP density, 4) available amenities for the GPs’ families, or 5) a previous experience as an intern, locum or associate in the area; it was possible to choose several items simultaneously.

Finally, we exploited a previous edition of the survey (Round 3 of the third national panel of French self-employed GPs, December 2015 to March 2016) to construct a dummy variable indicating the presence of a MGP in a *département* in 2013.

### Statistical analysis

To correct for possible systematic non-response bias in our subsample, we used weights to match the nationwide GP population for the four main stratification variables: age, gender, workload and GP density. When missing data occurred for some variables, they were included in the analysis and treated as a separate ‘missing’ category.

We defined a set of dependent variables regarding chronic care management (see Additional file 1 for a detailed description of the questionnaire): (1) a dummy variable reporting estimated variation in the number of weekly visits related to complications of chronic diseases (“Over the past week, what was the change in the frequency of visits related to complications of previously stable chronic diseases, compared to a typical week before the epidemic of Covid-19?”), as well as (2) a dummy variable indicating whether the GP makes a proactive effort to contact her chronic patients herself (“To address the current care needs of your most at-risk chronic patients, do you take an active approach to contacting them (by phone or other means of communication)?”).

We use probit regressions to estimate the following model:

$${Y}_i={\alpha}_i+{\beta}_1\ {MGP}_i+{\beta}_2\ Covid19\ {indicators}_i+{\beta}_3\ {Control\ variables}_i+{\varepsilon}_i$$

where

- *Y*_*i*_ is one of the dependent variables described above,
- *MGP*_*i*_ is a dummy variable indicating practicing in MGP,
- *Covid-19 indicators*_*i*_ include the indicator variable of the intensity of the pandemic presented above, as well as GPs’ perceptions regarding the severity of Covid-19 (from 0 “not at all severe” to 10 “extremely severe”) and their estimation of the percentage of the French population that would be contaminated by Covid-19 by the end of 2020 (less than 50, 50 to 75%, more than 75% of the population),
- and the control variables include GP i’ personal and professional characteristics: gender, age (in tertiles), workload (in tertiles), as well as a dummy variable indicating whether the practice is located in the area with the lowest (first decile) GP density in 2018 or not.

To address the possible endogeneity of choice of practicing in MGP, we estimate the bivariate probit model, since it is suited to simultaneously estimating both equations with binary outcome variables:

$$\left\{\begin{array}{c} MG{P}_i={\alpha}_i+{\gamma}_1\ {women}_i+{\gamma}_2\ {age}_i+{\gamma}_3\ {workload}_i+{\gamma}_4\ lowest\ GP\ {density}_i+\\ {}{\gamma}_5\ Pioneer\ d{\acute{e}}{partement}_i+{\delta}_i\ {Motivation}_i+{\varepsilon}_{i1}\\ {}{Y}_i={\alpha}_i+{\beta}_1\ {MGP}_i+{\beta}_2\ Covid19\ {indicators}_i+{\beta}_3\ {Control\ variables}_i+{\varepsilon}_{i2}\end{array}\right.$$

where the equation related to practicing in MGP contains several variables that might influence the GPs’ choice, but are unlikely to have an impact on chronic care management strategies during the sanitary crisis:

- *Pioneer département*_*i*_ is a dummy variable indicating practices located in a *département* that had adopted MGP early (before 2013),
- *Motivation*_*i*_ is a set of dummy variables regarding the selection criteria prior to the choice of the current practice location (healthcare services available, possibility of creating or joining a group practice, search for an area with low GP density, available amenities, or previous medical studies (a previous experience, e.g., internship, in the area)).

These two sets of instruments relate to the past behaviour of GPs or the behaviour of peers in the same *département* before 2013 (we use the term ‘instrument’ to emphasise the fact that these variables allow the identification of the model, even if the estimated model is not using a two-step procedure). This strengthens the fulfilment of the exclusion condition. As bivariate probit models do not allow testing for overidentification restrictions, we follow the procedure described in Wooldridge [16]. Using a linear probability model, we calculate the fitted value for MGP_i_. Next, we estimate the model described above by a Two-Stage Least Squares using the fitted value for MGP_i_. Having this adapted framework at our disposal, we were able to test the statistical properties of the instrumental variables using the Sargan overidentification test (The instruments are considered valid when they are correlated with the explanatory variables and uncorrelated with the error term. An instrument is considered weak when there is significant correlation between the instrument and the explanatory variable, but with a low value for the correlation coefficient). For both models, we calculate the marginal effects (on predicted probabilities) for GPs practicing outside MGP, outside the most affected *départements*, male, youngest, with lowest workload, those not practicing in lowest GP density areas.

All analyses were conducted with Stata 14 (StataCorp. College Station, Texas).

## Results

Out of the 1191 GPs that responded to the survey, almost 40% were female and 34% were younger than 50 years old (Table 1). Six percent were practicing in an area with the lowest GP density, 13% practiced in a MGP (this proportion was significantly higher among the youngest GPs: 44%). Twelve per cent of GPs were located in the *départements* most affected by Covid-19 (9% in MGP). The average perceived severity of Covid-19 was around 7.8 (out of 10). Forty-four percent of GPs estimated that by the end of 2020 less than half of the French population would be contaminated by Covid-19 and 13% believed that 75% or more would be contaminated.

**Table 1:** Descriptive statistics, French self-employed general practitioners (*n* = 1191)

%	Total	in MGP	not in MGP
*Observations*	*1191*	*254*	*839*
**GPs’ personal and professional characteristics**			
**Female**	39.18	38.90	40.46
**Age**			
< 50 years old	33.64	43.62***	33.98
50–59 years old	40.48	44.15***	39.14
60 years old or more	25.88	12.24***	26.87
**Workload**			
Q1	23.40	17.58	24.41
Q2-Q3	50.70	57.82	50.10
Q4	25.90	24.60	25.49
**Lowest GP density (1st decile), 2018**	6.31	9.14*	6.08
**Multiprofessional group practice (MGP)**	12.65	–	–
*missing observations*	*98*	–	–
**COVID-19 indicators**			
The most affected *départements*	12.25	8.59**	14.11
Estimated severity [0;10] (mean, standard error in brackets)	7.78 (0.09)	7.65 (0.13)	7.79 (0.11)
*missing observations*	*45*	*33*	*9*
**Estimated share of population contaminated by the end of 2020**			
< 50%	44.24	42.19	46.54
50–75%	42.46	40.94	40.16
75% or more	13.31	16.87	13.30
*missing observations*	*362*	*56*	*279*
**GPs’ practices during the pandemic**			
Number of consultations related to complications of chronic diseases dropped during the week before the survey compared to a “typical” week before the pandemic	69.74	61.52	69.60
*missing observations*	*103*	*24*	*79*
Pro-active in contacting chronic patients	50.16	61.65**	48.11
*missing observations*	*59*	*12*	*48*

Source: DREES, ORS and URPS Provence-Alpes-Côte d’Azur and Pays de la Loire, 4ème Panel d’observation des pratiques et des conditions d’exercice en médecine générale de ville

Note: weighted data

*GPs* general practitioners, *MGP* multi-professional group practices

^*^p < 0.1

^**^p < 0.05

^***^p < 0.01 (chi-squared test)

Seventy percent of GPs estimated that, compared to their “typical” week before the pandemic, the frequency of visits related to complications of chronic diseases had dropped. Half of GPs declared that they contacted their chronic patients themselves (62% in MGP).

Regarding the frequency of visits related to complications of chronic diseases (Table 2), the estimated value of the “rho” coefficient first advocates for the use of the bivariate probit model; a result that confirms the impact of the self-selection bias for GPs in MGP. Beyond these technical considerations, in this regression (column 2), GPs in MGP were less likely to experience a drop in these visits (− 45.3%).

**Table 2:** Factors associated with general practitioners’ practices during the lockdown (*n* = 1191)

Marginal effects	Number of visits related to complications of chronic diseases dropped during the week before the survey compared to a “typical” week before the pandemic		Pro-active in contacting chronic patients	
	Probit	Bivariate probit	Probit	Bivariate probit
**Multiprofessional group practice (MGP)**	−0.0199	−0.4527***	0.1344**	0.3636***
	(0.0564)	(0.0975)	(0.0579)	(0.1293)
**The most affected*départements***	0.0418	0.0743	−0.0817	−0.0662
	(0.0731)	(0.0534)	(0.0919)	(0.0952)
**Perceived severity [0;10]**	−0.0023	− 0.0076	0.0146	0.0176
	(0.0118)	(0.0098)	(0.0138)	(0.0137)
**Estimated share of population contaminated by the end of 2020 (ref. < 50%)**				
50–75%	−0.0977	−0.0527	0.1887***	0.1500**
	(0.0631)	(0.0507)	(0.0618)	(0.0636)
75% or more	−0.0466	−0.0223	0.1532**	0.1268*
	(0.0723)	(0.0593)	(0.0751)	(0.0759)
**Female**	0.0083	−0.0186	0.0810	0.0948
	(0.0556)	(0.0482)	(0.0618)	(0.0634)
**Age (ref. < 50 years old)**				
50–59 years old	−0.0874	− 0.0951*	0.0489	0.0409
	(0.0605)	(0.0547)	(0.0648)	(0.0646)
60 years old or more	0.0590	0.0432	−0.0131	−0.0144
	(0.0659)	(0.0606)	(0.0811)	(0.0873)
**Workload (ref. Q1)**				
Q2-Q3	−0.0941	−0.0645	− 0.1294*	−0.1516**
	(0.0642)	(0.0534)	(0.0695)	(0.0720)
Q4	−0.0167	−0.0127	− 0.1248	−0.1114
	(0.0843)	(0.0719)	(0.0896)	(0.0907)
**Lowest GP density (1st decile)**	0.0763	0.0635	−0.1396**	−0.1464**
	(0.0501)	(0.0425)	(0.0663)	(0.0656)
**ρ (coefficient)**	–	0.8701***	–	−0.5758
		(0.2532)		0.4544
Observations	680	641	704	665

Source: DREES, ORS and URPS Provence-Alpes-Côte d’Azur and Pays de la Loire, 4ème Panel d’observation des pratiques et des conditions d’exercice en médecine générale de ville

Notes: Weighted data

Standard errors in brackets

Marginal effects calculated for: non-MGP, GPs not in most affected *départements*, male GPs, youngest GPs, with lowest workload, those not practicing in lowest GP density areas

Reading note: For GPs practicing in MGP, the probability of experiencing a drop in visits related to complications of chronic diseases is 1.99% lower (probit)/ 45.25% lower (bivariate probit) compared to those practicing outside MGP

The estimated value of the “rho” (ρ) coefficient represents the correlation coefficient between the residuals of each of the two equations. A “rho” coefficient statistically significantly different from zero advocates for the use of the bivariate probit model (simultaneous estimation of the two equations)

*GPs* general practitioners, *MGP* multi-professional group practices

^*^p < 0.1

^**^p < 0.05

^***^p < 0.01 (probit and bivariate probit regressions)

As far as the second behaviour in Table 2 is concerned: ‘pro-active in contacting chronic patients’, the rho coefficient of the bivariate probit estimation indicates, this time, an absence of effect of the self-selection into MGP and that the simple probit model is sufficient to give the proper estimate. In the results (column 3), GPs in MGP were more proactive in contacting their chronic patients (+ 13.4%). This exactly counteracts the impact of practicing in the lowest GP density area (− 14.0%). In addition, those who believed that Covid-19 would contaminate less than 50% of the population were less likely to contact their chronic patients.

As for the instruments used, Sargan test results indicated the validity of the instruments (*p* = 0.11 and 0.09 respectively), however the instruments were weak: the F statistic was below the commonly-used threshold of 10.

## Discussion

This study evaluated how GPs in MGP performed regarding chronic care management during the first French lockdown, compared to their counterparts practicing outside MGP. The vast majority of GPs reported that, compared to their “typical” week before the pandemic, the frequency of visits related to complications of chronic diseases had dropped. Only half of GPs declared having proactive behaviour, such as contacting their chronic patients. Our research assumption was that GPs in MGP would not experience the same patterns of negative impacts of the Covid-19 crisis. Taking into account the self-selection of GPs in joining a MGP, those in MGP were less likely to experience a drop in these visits and were more likely to adopt proactive behaviour.

In France, GPs are the cornerstone of the health system: in 2017, around 80% of patients had consulted a GP in the past 12 months [17]. The integration of care remains one of the current challenges of the French healthcare system. Despite a clear trend towards primary care integration, the 2019 French GP survey indicated that while 61% of respondents declared practicing in a group practice, 57% among them stated that their practice was composed exclusively of GPs [7]. Indeed, only 13% of GPs in our sample worked in MGP.

Creating integrated practices in primary care settings is an organizational adjustment strongly promoted by the French government, in order to improve access to care and quality of care, particularly for populations living in deprived areas [10, 18]. However, demonstrating this improvement is sometimes difficult. Many studies have mixed results and barely demonstrate a definite impact of integrated practices on the quality of care - quality is hard to document in family medicine [19–26]. Using the follow-up of chronic patients during lockdown as a criterion for quality of care, this study provides the opportunity to test whether quality of care was enhanced by this type of organizational adjustment during an unprecedented health crisis.

The continuous follow-up of chronic patients is a necessary condition for the quality of care. In times of pandemic, while most resources are allocated to fight Covid-19, it is crucial to ensure the continuity of care for those already vulnerable amongst the population such as chronic patients [27, 28].

Our findings are in line with both French [10] and international literature [29, 30] which report that GPs in integrated practices are “more productive”. Using our own data, we obtain that these GPs can indeed transform these labour-productivity gains to the benefit of the patient. This result is in line with our results, as long as we assume that the productivity gains, and the additional time resource they generate, could be equally transmitted to all sectors of the GPs’ medical activities, including chronic care management.

In times of sanitary crises, GPs in MGP are more likely to ensure continuity of care for their chronic patients than those outside MGP. This result is established while taking into account the selection effect that may occur from GPs in MGP, eliminating the main risk of false causal inference. This additional benefit for patients of GPs in MGP probably results from better organization, especially in terms of task division in group practices. For instance, it is quite common that MGP nurses are in charge of the follow-up of chronic patients. In addition, sanitary guidelines could be easier to implement in larger practices, e.g., they might have the possibility of receiving potentially infected patients in separate waiting rooms.

This research has both strengths and weaknesses. The main strength consisted in being able to demonstrate that the MGP organizational formula (the French way to obtain the integration of primary care) was also beneficial to patients, at least to those with chronic conditions, and not only to healthcare professionals - there are more results in the literature demonstrating that latter point, while only a few were really able to deal with patients’ outcomes [12]. In our view, the choice of the lockdown as an observation period was especially relevant, as it was a period of severe stress for the entire healthcare system, and one which gave us the opportunity to verify the good functioning of the system when presented with real challenges. The weaknesses were that the research design did not measure the patients’ qualitative outcomes directly, but some variables that remained rather indirect: the frequency of consultations and proactivity. We believe that, rather than a sufficient condition, we obtained a necessary condition for the qualitative improvement in chronic care management. Another weakness was that the use of a dummy variable, representing participation in MGP, cannot really capture the large and multidimensional panel of care integration, with for example various forms of cooperation between multidisciplinary specialities. However, this is a clear institutional cut-off to class GPs and, therefore, this has added value for the public policy assessment.

We have obtained encouraging results on the added value of MGP during COVID-19. These results can provide recommendations both for the short term and for the long term management of chronic patients. In the short term, a solution could be to appoint secretaries or medical assistants (as introduced by national collective agreement in 2019) in charge of the follow-up of chronic patients. In the long run, it advocates for further development of integration in primary care all across the national territory. However, we should recognize that, beyond these casual observations, further research is needed to demonstrate the added value of integrated practices (at least this French experience of “multi-professional primary care group practices”) once the COVID-19 pandemic setup is over.

## Supplementary Information

**Additional file 1.** “Covid-19” online questionnaire, fourth national panel of French self-employed general practitioners (April, 9 to April, 21, 2020).

## Data Availability

The dataset used during the current study comes from the fourth national panel of French self-employed GPs (https://drees.solidarites-sante.gouv.fr/sources-outils-et-enquetes/00-le-panel-dobservation-des-pratiques-et-des-conditions-dexercice-en) and is available on request from the Directorate for Research, Studies, Assessment and Statistics (DREES), French Ministry of Health. All authors had permission to access and use the dataset.

## Abbreviations

GPs: General practitioners
MGP: Multi-professional group practice
