# Supplementary material for: United, can we be stronger? Did French general practitioners in multi-professional groups provide more chronic care follow-up during lockdown?
Source: BMC Health Serv Res. 2022 Apr 19;22:519. doi: 10.1186/s12913-022-07937-z (PMC9016683; doi:10.1186/s12913-022-07937-z)
Supplement: Supplementary file 1 — Additional file 1. “Covid-19” online questionnaire, fourth national panel of French self-employed general practitioners (April, 9 to April, 21, 2020). [file 12913_2022_7937_MOESM1_ESM.docx]

# Additional File 1. “Covid-19” online questionnaire, fourth national panel of French self-employed general practitioners (April, 9 to April, 21 2020)

Note: The full questionnaire consisted of 25 questions. Below are listed the questions used for this work. General practitioners’ personal and professional characteristics were collected at inclusion in the panel.

*[Introduction “…”]*

- Last week, what was the change in the frequency of consultations for each of the following reasons compared to an ordinary week before the outbreak of Covid-19 epidemic? Reasons related to complications of previously stable chronic diseases
  - Increased by more than 50%
  - Increased by less than 50%
  - As frequent as before
  - Decreased by less than 50%
  - Decreased by more than 50%
  - You do not do this type of consultation
  - Refusal
  - Don't know
- To address the current care needs of your most at-risk chronic patients, do you take an active approach to contacting them (by phone or other means of communication)?
  - Yes
  - No
  - Refusal
  - Don't know
- What is your opinion of the medical severity, relative to the general population, of Covid-19 on a scale of 0 to 10?

/_ _ / (integer from 0 to 10/refusal/don’t know)

*Give a score on the scale from 0 (‘not at all severe’) to 10 (‘extremely severe’). The intermediate scores allow you to refine your judgement.*

- In your opinion, out of 100 people living in France, how many will have been contaminated by the coronavirus (Covid-19) by the end of the year?

  /_ _ / (integer from 0 to 100/refusal/don’t know)

*[Closing sentence: “That is the end of the questionnaire. Thank you very much for your contribution, which will be of great help to us in our survey.”]*
